# Supplementary material for: Structure-guided discovery of highly efficient cytidine deaminases with sequence-context independence
Source: Nat Biomed Eng. 2024 Jun 3;9(1):93–108. doi: 10.1038/s41551-024-01220-8 (PMC11754093; doi:10.1038/s41551-024-01220-8)
Supplement: Supplementary file 1 — Supplementary figures. [file 41551_2024_1220_MOESM1_ESM.pdf]

nature biomedical engineering

Supplementary information https://doi.org/10.1038/s41551-024-01220-8

Structure-guided discovery of highly efficient cytidine deaminases with sequence-context independence

In the format provided by the authors and unedited

Contents

Supplementary figures

1 Clustering of candidate cytidine deaminases
2 Clustering of candidate cytidine deaminases by hierarchical clustering
3 The design of CBEs and sgRNAs
4 Editing efficiencies of candidate deaminase-derived CBEs
5 Editing windows of candidate deaminase-derived CBEs
6 Editing properties of candidate deaminase-derived CBEs from cluster #147
7 Editing windows of the top eight deaminase-derived CBEs
8 Editing efficiency of CD0208-derived CBE
9 Amino acid residues in CD0208 potentially interacted with ssDNA
10 Off-target effects of CD0208 variants-derived CBEs
11 Editing properties of CD0208 variants-derived CBEs
12 CD0208^{P52A} CBE introducing nonsense mutations in the *Tyr* gene in mouse N2A cells
13 Editing windows of CD0208^{P52A} CBE at 11 target sites in the *Tyr* gene in mouse N2A cells
14 Off-target effects of CD0208^{P52A}-derived CBE
15 CD0208^{P52A} CBE inducing nonsense mutations in three multi-copy genes in mESCs
16 CD0208^{P52A} CBE introducing nonsense mutations in muti-copy genes in porcine cell PK-15
17 Editing windows of CD0208 ^{P52A}-nSaCas9 CBE at seven target sites in HEK293T cells
18 Editing windows of CD0208^{P52A} nSpCas9-NG CBE at eight target sites in HEK293T cells
19 Editing windows of CD0208^{P52A}-nSpCas9, and CD0208^{P52A}-dCpf1 CBEs at endogenous loci in HEK293T cells
20 CD0208^{P52A} CBE introducing nonsense mutations in *Hpd* and *Pcsk9* genes in N2A cells

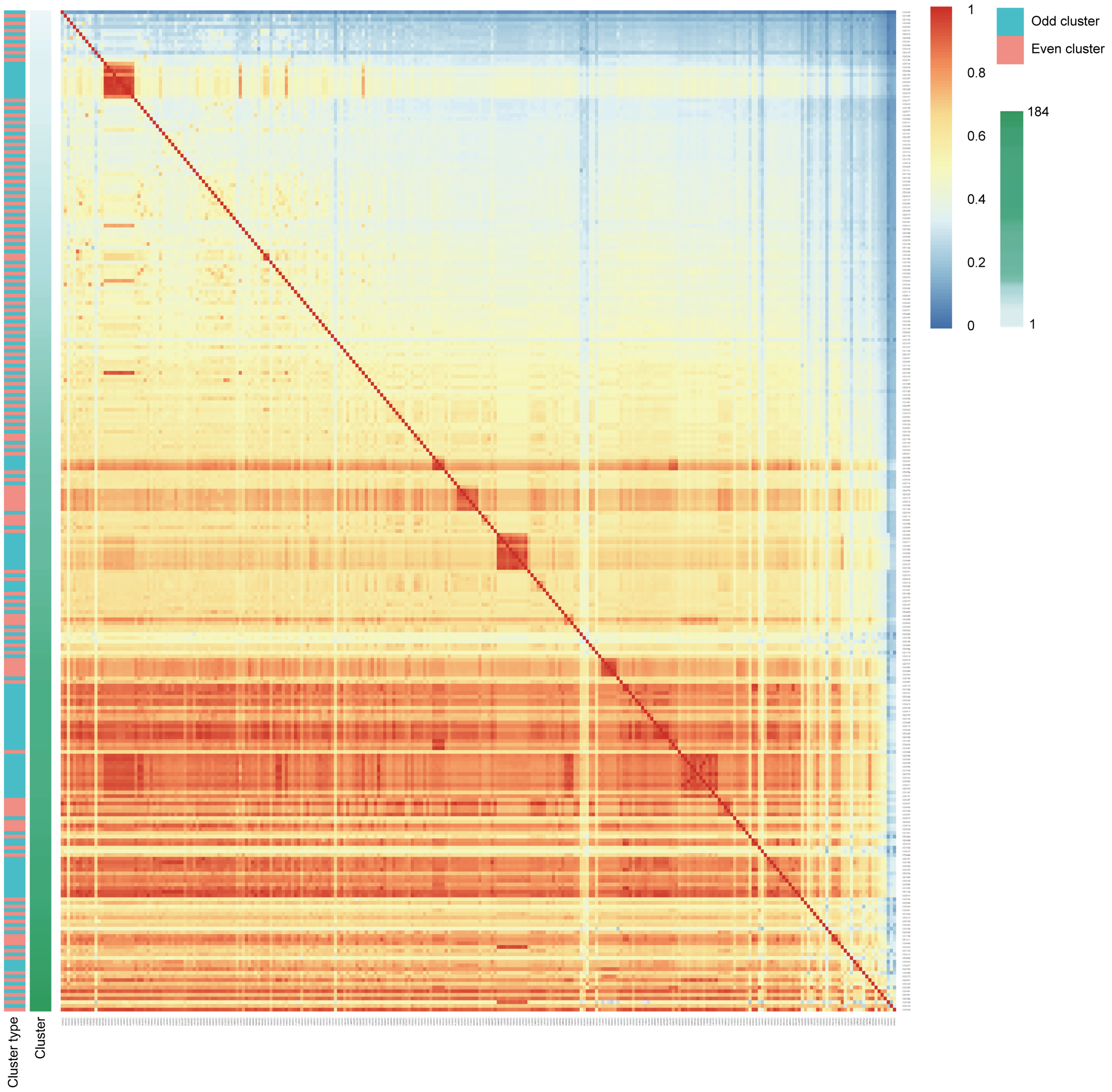

Supplementary Fig. 1 | Clustering of candidate cytidine deaminases. Clustering the 272 selected deaminases based on 3D structural similarity. The red-to-blue heatmap represents the level of structural similarity. The green-to-white gradient indicates the cluster number. Odd clusters (such as, clusters #1, #3, #5, etc.) are marked in blue; even clusters (such as, clusters #2, #4, #6, etc.) are marked in red.

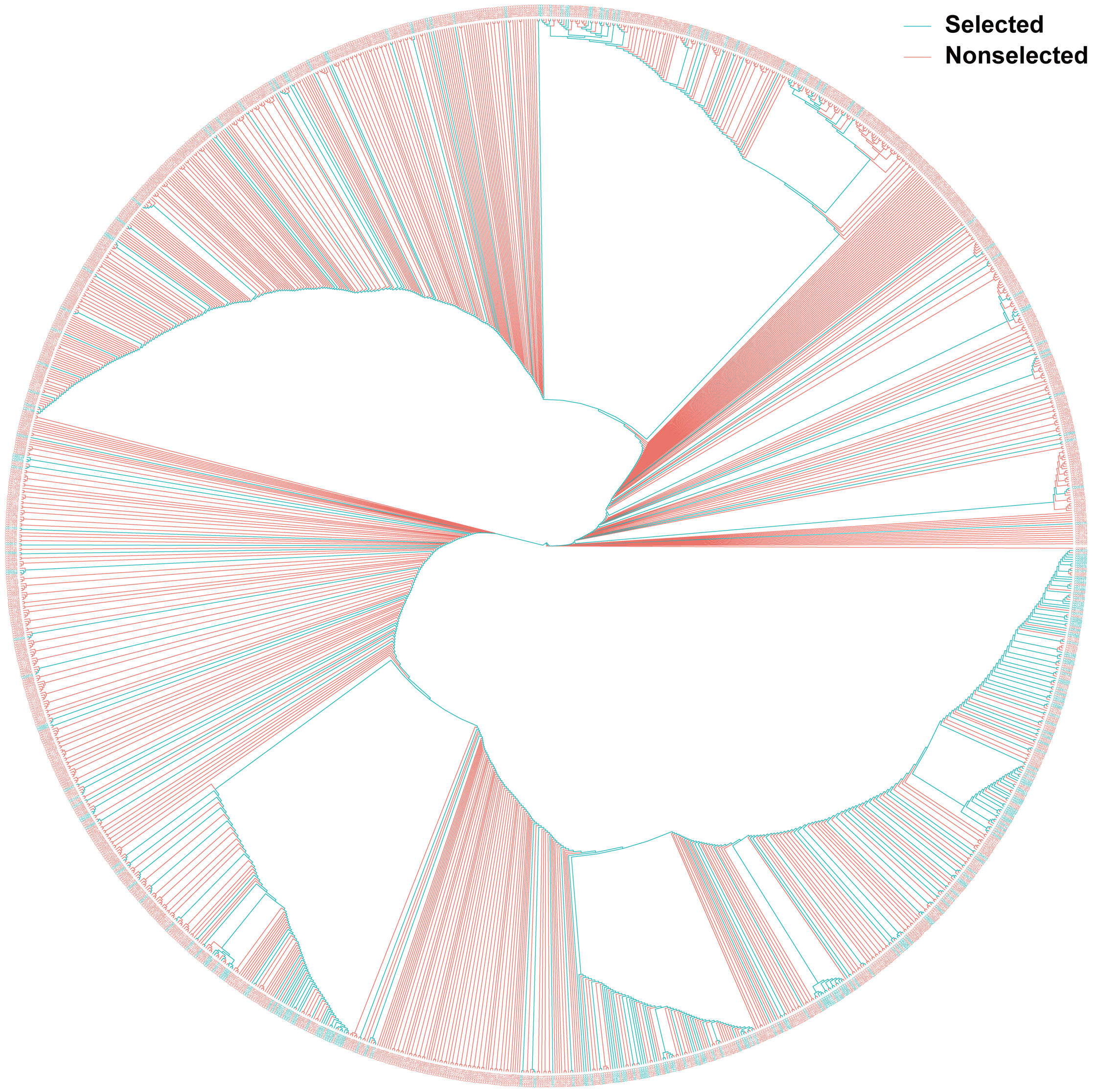

Supplementary Fig. 2 | Clustering of candidate cytidine deaminases by hierarchical clustering. Phylogenetic analysis of cytidine deaminases according to predicted 3D structure by hierarchical clustering. The green colors represent the 272 deaminases selected from 184 clusters categorized by partitional clustering, and the red plus green colors display a total of 1483 deaminases. The CD0847 from the species Latimeria_chalumnae was selected as an outgroup.

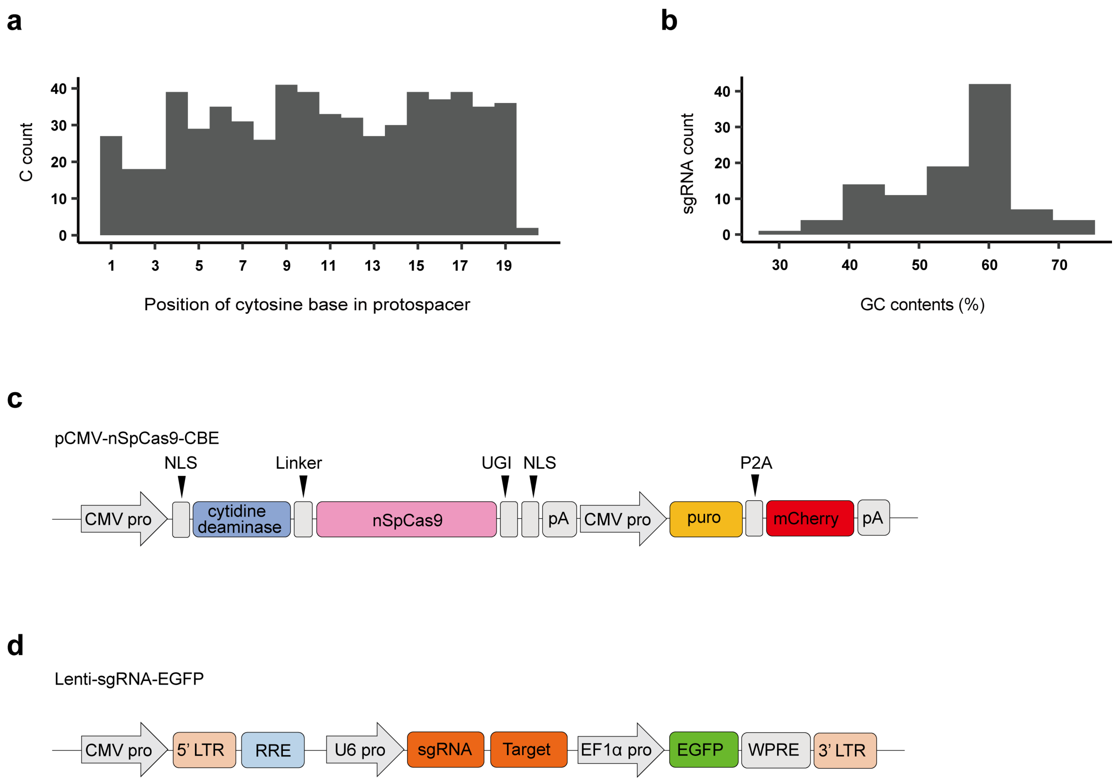

Supplementary Fig. 3 l The design of CBEs and sgRNAs. a, Distribution of cytosine bases in the protospacer in the 102 sgRNA-target library. **b**, GC content analysis of a 102 sgRNA-target library. **c**, Schematics of CBE vector backbone in a 102 sgRNA-target library. **d**, Schematics of sgRNAs of the Lentiviral vector.

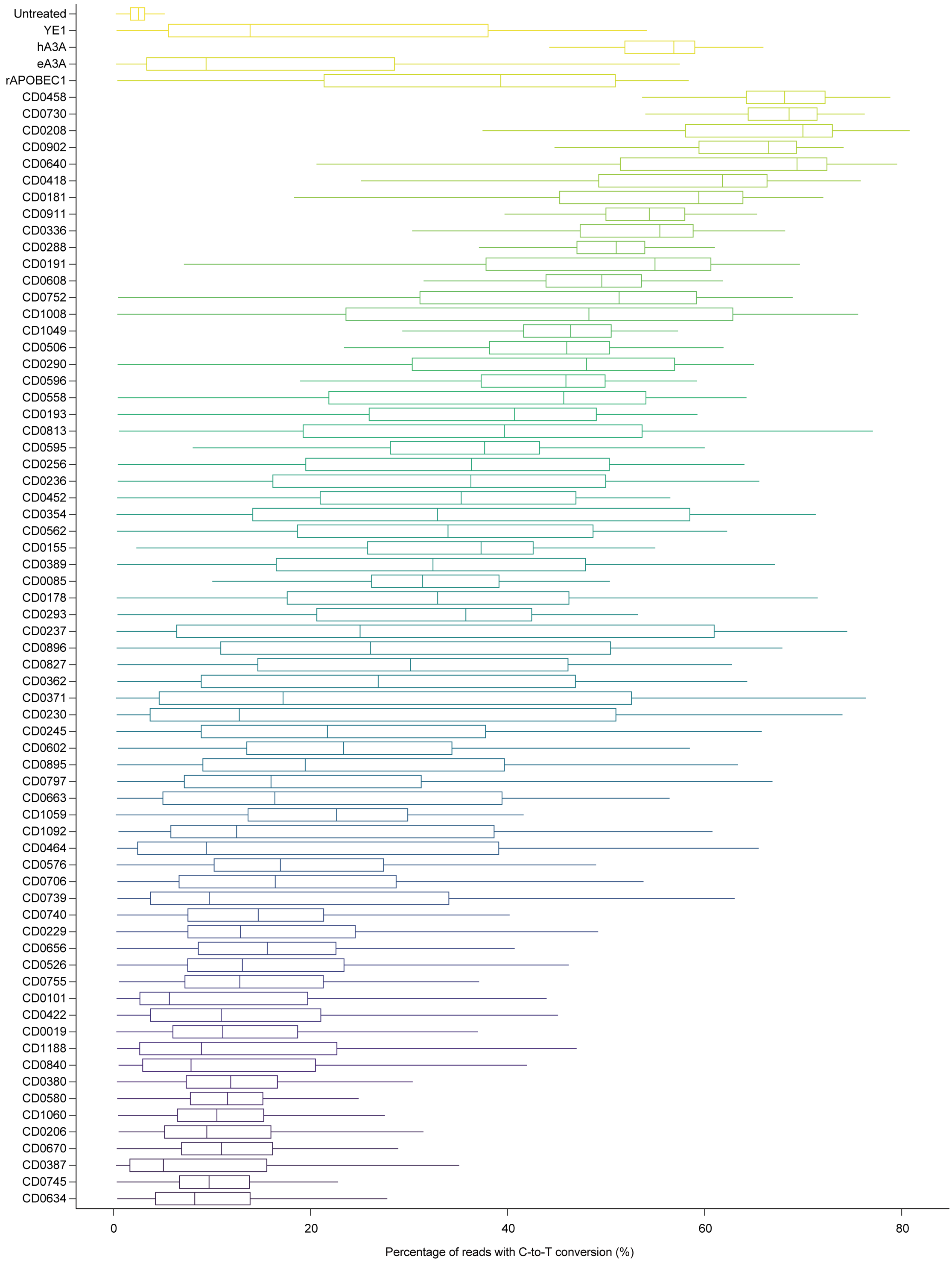

Supplementary Fig. 4 | Editing efficiencies of candidate deaminase-derived CBEs. C-to-T editing efficiencies for the discovered candidate and four well-characterized deaminases (hA3A, rAPOBEC1, YE1, and eA3A) -based CBEs using a 102 sgRNA-target library via deep sequencing. The center lines indicate the median, while the bottom and top lines of the boxes represent the first quartile and third quartile of the editing efficiencies at 102 sgRNA-target sites, respectively. Tails extend to the minimum and maximum values.

	1	2	3	4	5	6	7	8	9	10	11	12	13	14	15	16	17
Untreated	0.07	0.14	0.21	0.32	0.34	0.32	0.21	0.12	0.1	0.07	0.07	0.05	0.04	0.04	0.03	0.03	0.02
eA3A	0.24	0.44	1.65	12.9	13.38	12.83	6.81	3.35	1.21	0.54	0.41	0.44	0.24	0.46	0.08	0.04	0.05
YE1	0.05	0.61	7.38	23.63	24.56	17.65	3.56	1.28	0.2	0.13	0.07	0.11	0.05	0.07	0.09	0.03	0.05
rAPOBEC1	2	7.44	17.06	34.84	34.37	32.5	20.81	10.39	6.65	4.24	3.92	2.12	1.25	1.34	1.25	0.35	0.41
hA3A	28.68	39	43.47	47.63	48.15	47.71	43.13	37.21	32.23	23.14	19.33	19.13	17.72	13.61	6.06	3.28	1.18
CD0458	35.34	51.96	58.1	62.56	62.82	61.45	57.61	51.7	42.86	27.55	22.68	17.81	15.04	15.36	5.56	3.64	1.36
CD0730	31.68	47.51	54.43	59.61	59.93	58.32	55.49	46.46	37.2	24.44	21.07	16.39	14.87	14.46	5.15	2.52	1.21
CD0208	22.42	48.49	60.43	63.37	61.96	53.8	34.79	16.24	9.29	9.72	5.57	3.17	2.42	0.6	0.57	0.34	0.17
CD0902	25.84	47.75	55.3	60.08	58.99	57.63	50.82	42.5	23.48	17.48	19.98	15.11	14.68	6.47	4.11	1.45	0.36
CD0640	23.31	48.1	58.26	63.03	61.4	52.61	34.4	14.64	7.35	7.36	3.81	1.84	1.85	0.37	0.39	0.29	0.17
CD0418	29.87	40.65	49.87	54.63	55.17	49.69	50.52	38.52	19.46	17.76	12.57	6.81	3.42	2.7	2.18	3.88	1.05
CD0181	34.97	47.72	50.16	52.91	53.59	42.64	42.55	41.11	30.66	26.56	22.31	8.23	2.38	1.14	0.83	0.99	0.28
CD0911	10.47	29.02	43.11	47.49	46.69	41.18	27.06	22.35	22.33	26.96	24.12	16.37	9.48	7.53	3.49	1.39	0.5
CD0336	33.24	42.76	45.75	49.05	47.47	38.12	37.57	37.24	28.76	24.74	20.21	7.57	2.43	0.86	0.71	0.97	0.34
CD0288	24.1	37.66	42.74	45.5	45.82	45.9	42.67	36.68	22.69	19.48	22.71	17.54	14.32	8.7	4.29	1.39	0.31
CD0191	29.94	40.97	41.26	43.53	45.5	29.73	27.84	17.79	10.78	14.31	10.38	4.58	4.17	1.89	1.48	3.09	1.1
CD0608	24.96	35.2	41.17	44.52	44.17	43.49	39.99	35.6	30.06	22.65	16.05	7.86	3.27	2	1.72	0.76	0.35
CD0752	22.73	36.08	37.96	41.84	38.01	34.14	33.95	24.46	11.71	8.43	5.6	3.05	1.81	1.78	3.1	2.88	0.77
CD1049	15.69	22.77	26.45	31.86	35.53	33.49	31.21	24.38	23.35	15.38	10.39	8.81	9.06	8.11	3.68	2.39	1.31
CD1008	18.71	34.65	37.24	39.58	35.07	30.45	30	20.83	8.75	6.38	4.41	2.19	1.43	1.19	2.47	2.78	0.73
CD0506	8.34	20.83	33.04	38.36	36.89	28.36	18.27	15.66	18.74	23.72	23.37	19.3	7.36	3.42	1.92	0.93	0.51
CD0290	12.71	18.24	27.39	37.04	40.32	32.68	26.75	14.87	12.23	8.25	6.59	5.13	5.27	4.56	0.97	0.8	0.5
CD0596	9.02	14.55	18.45	25.94	30.88	30.61	30.04	30.02	35.45	33.45	25.74	18.51	12.37	11.39	2.06	0.71	0.28
CD0558	4.69	17.06	29.89	43.03	41.27	33.11	20.32	10.18	2.55	1.11	0.55	0.31	0.2	0.27	0.22	0.19	0.12
CD0193	16.63	28.91	30.18	36.87	37.01	31.05	28.7	16.14	8.75	6.68	4.26	2.47	1.31	1.5	1.64	1.56	0.61
CD0813	19.15	31.08	34.77	33.29	32.15	30.14	26	17.34	15.19	15.09	9.21	5.13	0.98	0.65	0.66	0.35	0.22
CD0595	3.24	10.15	23.04	32.71	33.57	31.26	22.99	13.47	9.04	6.79	7.14	3.63	1.8	2.46	1.38	0.73	0.59
CD0256	27.93	39.85	35.06	35.08	31.66	22.34	16.11	4.38	1.3	0.26	0.11	0.15	0.11	0.2	0.29	0.1	0.12
CD0236	1.81	5.36	18.04	34.78	35.26	24.13	15.72	11.89	4.34	9.66	4.84	2.7	0.8	0.24	0.16	0.13	0.11
CD0354	0.24	3.95	15.98	37.71	38	31.02	12.38	3.65	0.51	0.47	0.32	0.18	0.05	0.13	0.11	0.08	0.09
CD0452	11.78	17.22	18.9	27.23	29.25	28.25	29.14	23.04	17.5	17.95	14.37	9.72	5.35	0.95	0.65	0.53	0.22
CD0562	5.66	11.13	17.59	28.52	32.77	25.75	19.54	8.87	6.47	4.16	3.49	2.43	2.47	2.57	0.56	0.43	0.28
CD0155	5.6	12.82	21.8	29.39	30.66	25.73	19.3	11.89	8.66	4.4	3.1	1.86	1.38	1.52	0.67	0.43	0.33
CD0389	0.71	2.39	8.09	17.45	23.14	17.07	14.89	14.08	19.65	19.5	16.49	10.68	6.26	2.17	1.44	0.42	0.08
CD0085	13.02	21.87	24.79	29.28	30.06	26.76	23.63	17.03	14.44	9.42	7.22	4.6	2.58	4.38	5.97	2.19	0.61
CD0178	13.22	26.6	27.49	30.85	27.99	23.31	19.88	12.58	5.48	3.95	2.1	1.36	0.71	0.75	1.61	0.83	0.31
CD0237	0.11	3.55	15.63	36.17	33.85	24.75	8.33	3.5	0.41	0.55	0.21	0.28	0.08	0.1	0.09	0.08	0.1
CD0293	29.25	34.5	33.41	33.13	29.83	25.99	21.22	7.47	4.49	2.38	2.01	0.77	0.33	0.14	0.4	0.32	0.17
CD0896	9.42	13.07	13.98	24.86	27.39	12.08	8.38	6.89	3.29	6.7	5.3	1.2	0.49	0.35	0.25	0.27	0.16
CD0827	0.38	4.16	9.58	18.27	25.83	18.49	17.27	6.51	5.37	7.86	9.35	4.75	3.36	1.86	0.3	0.19	0.12
CD0362	2.55	3.98	10.52	24.95	28.29	22.95	13.39	8.15	4.76	3.64	2.99	1.54	0.54	0.56	0.27	0.1	0.14
CD0371	0.28	2.11	8.89	25.68	29.02	24.27	8.18	3.21	0.55	0.54	0.22	0.28	0.13	0.14	0.14	0.08	0.12
CD0230	0.08	1.41	9.08	24.17	26.56	21.97	5.72	1.91	0.27	0.25	0.11	0.13	0.05	0.09	0.07	0.04	0.07
CD0602	2.39	5.07	9.27	13.24	16.5	13.22	13.68	14.22	17.29	19.74	19.73	13.91	8.45	5.86	4.38	1.04	0.17
CD0245	0.82	6	8.68	22.52	23.59	15.28	8.6	2.57	1.15	0.93	0.61	0.25	0.22	0.13	0.2	0.07	0.06
CD0895	1.14	2.41	14.24	25.01	17.98	8.72	2.87	1.35	1.99	4.09	2.06	0.81	0.42	0.16	0.24	0.15	0.19
CD0663	0.33	1.47	7.21	21	20.69	16.93	9.45	5.98	2.1	1.54	0.88	0.71	0.25	0.26	0.28	0.13	0.15
CD0797	1.34	2.52	3.64	21.38	24.44	9.85	5.2	2.04	0.63	1.11	1.03	1.24	2.11	1.83	0.33	0.15	0.13
CD1059	0.61	1.27	3.74	13.62	18.24	12.81	11.54	5.66	6.22	9.5	11.16	9.54	6.57	1.01	0.14	0.12	0.07
CD0464	0.2	0.58	5.66	20.63	23.29	18.38	7.47	2.75	0.76	0.61	0.32	0.16	0.09	0.14	0.11	0.03	0.07
CD1092	0.31	2.2	10.62	21.29	23.44	18.74	5.47	1.72	0.51	0.39	0.25	0.28	0.19	0.19	0.16	0.09	0.12
CD0576	5.56	11.93	15.56	16.13	13.07	10.4	9.24	5.28	1.69	1.37	0.77	0.36	0.3	0.36	0.58	0.58	0.15
CD0706	0.65	0.48	1.5	10.11	14.5	13	9.05	4.99	6.59	4.79	3.33	2.24	2.45	3.71	0.2	0.09	0.1
CD0739	0.26	1.85	6.82	16.96	19.31	15.5	4.88	1.5	0.32	0.27	0.16	0.16	0.12	0.1	0.11	0.06	0.12
CD0740	0.25	0.6	2.39	3.7	6.27	6.4	4.82	3.3	9.01	13.14	8.8	2.07	1.1	0.63	0.14	0.07	0.08
CD0229	0.59	6.53	10.65	6.79	5.19	3.83	3.5	4.3	3.13	5.07	4.28	2.02	1.16	1.21	2.58	0.85	0.39
CD0526	3.98	11.19	8.31	5.04	5.99	5.15	9.06	4.56	3.52	2.53	1.76	1.77	0.48	0.71	2.58	2.02	0.85
CD0656	5.07	6.59	4.32	8.06	10.54	9.99	12.38	10.45	6.18	9.2	6.36	3.98	2.68	0.86	0.71	0.65	0.32
CD0755	0.12	0.22	0.35	2.88	4.16	3.27	1.97	2.28	6.25	12.97	12.27	6.26	2.99	0.57	0.09	0.11	0.13
CD0422	0.13	0.48	1.05	6.51	9.58	9.36	7.36	3.55	3.57	2.37	2.33	1.57	1.78	2.66	0.1	0.06	0.06
CD0101	0.08	1.41	2.29	12.45	15.67	4.22	2.11	0.31	0.8	0.5	0.25	0.17	0.07	0.06	0.05	0.06	0.06
CD0019	8.77	12.22	10.62	7.72	8.82	3.51	3.47	1.32	0.93	0.54	0.42	0.42	0.38	0.44	0.71	0.86	0.52
CD1188	0.03	0.38	4.82	14.14	11.86	7.79	2.19	2.72	0.23	0.23	0.03	0.04	0.04	0.03	0.07	0.04	0.06
CD0840	0.36	1.62	4.72	10.58	12.02	7.75	2.38	0.84	0.17	0.18	0.12	0.17	0.15	0.1	0.14	0.12	0.14
CD0380	1.01	1.81	1.96	7.71	10.43	8.07	7.3	4.79	2.93	2.52	2.39	1.06	0.49	0.13	0.11	0.05	0.07
CD0580	0.78	2.3	3	8.45	8.12	6.6	4.58	3.53	2.46	2.94	3.33	2.67	1.01	0.53	0.15	0.06	0.07
CD0206	0.24	0.58	0.4	0.93	4.23	3.49	5.2	5.45	3.95	6.13	4.88	2.49	3.32	0.56	0.17	0.09	0.07
CD1060	5.98	6.59	8.67	10.33	9.75	8.21	7.19	1.77	0.25	0.14	0.04	0.14	0.14	0.12	0.1	0.1	0.13
CD0670	0.74	1.46	1.79	6.31	9.58	7.26	6.37	4.7	2.74	2.69	2.26	1.36	0.44	0.07	0.09	0.08	0.11
CD0387	0.06	0.67	2.29	3.96	8.32	4.06	3.01	0.47	0.77	4.74	3.11	1.1	0.77	0.04	0.05	0.08	0.05
CD0745	0.19	0.28	0.45	1.43	3.22	3.42	2.93	1.35	2.35	6.87	7.8	4.66	3.1	1.52	0.35	0.09	0.08
CD0634	3.08	10.2	8.16	5.45	4.46	3.05	2.84	1.19	0.79	0.2	0.1	0.07	0.06	0.1	0.24	0.29	0.11

Position of cytosine base in protospacer

Editing efficiency (%): 20, 40, 60

Supplementary Fig. 5 I Editing windows of candidate deaminase-derived CBEs. Editing windows for the cytidine deaminase-derived CBEs and four well-characterized deaminases (hA3A, rAPOBEC1, YE1, and eA3A) in a 102 sgRNA-target library via deep sequencing. The data represents the mean of four independent experiments.

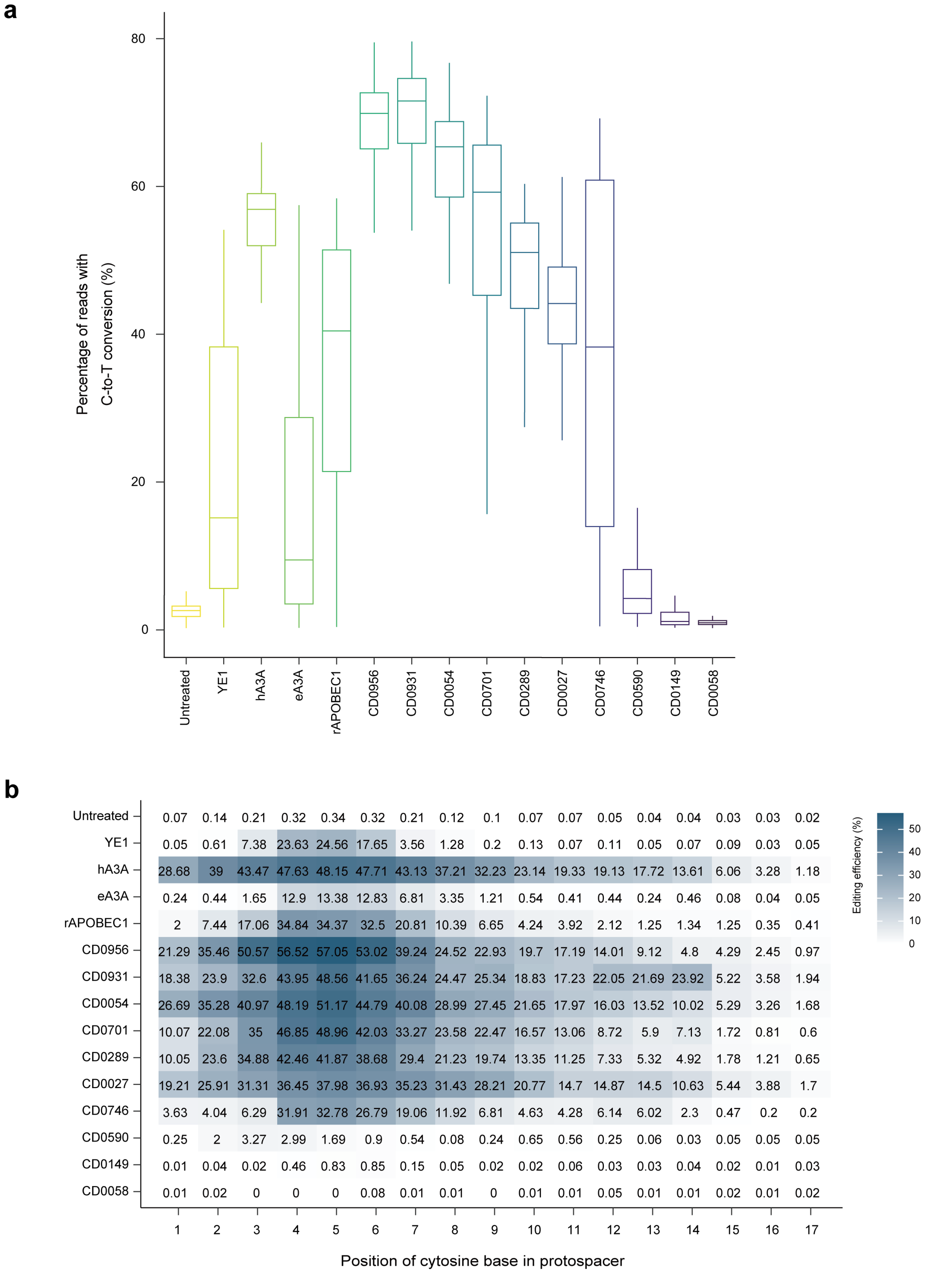

Supplementary Fig. 6 l Editing properties of candidate deaminase-derived CBEs from cluster #147. a, Average editing efficiencies of 10 cytidine deaminases from cluster #147 in a 102 sgRNA-target library. The center lines indicate the median, while the bottom and top lines of the box represent the first quartile and third quartile of the editing efficiencies at 102 sgRNA-target sites, respectively. Tails extend to the minimum and maximum values. **b**, The editing windows of 10 cytidine deaminases from cluster #147 in a 102 sgRNA-target library. Four well-characterized deaminases, hA3A, rAPOBEC1, YE1, and eA3A, were used as controls. The data represents the mean of four independent experiments.

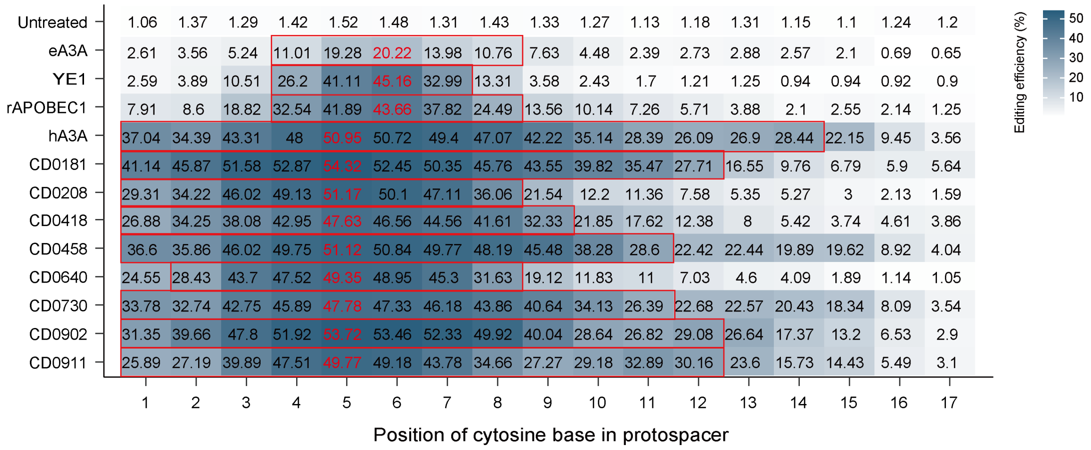

Supplementary Fig. 7 | Editing windows of the top eight deaminase-derived CBEs. Editing windows of CBEs containing the eight top deaminases (CD0458, CD0730, CD0208, CD0902, CD0640, CD0418, CD0181, and CD0911) and well-characterized deaminases (hA3A, rAPOBEC1, YE1, and eA3A) in an 11,868 sgRNA-target library through deep sequencing analysis. The red numbers represent the highest editing efficiencies for the cytosine deaminases, and the red boxes indicate the editing windows. The data represents the average editing efficiency at 11,868 sgRNA-target sites.

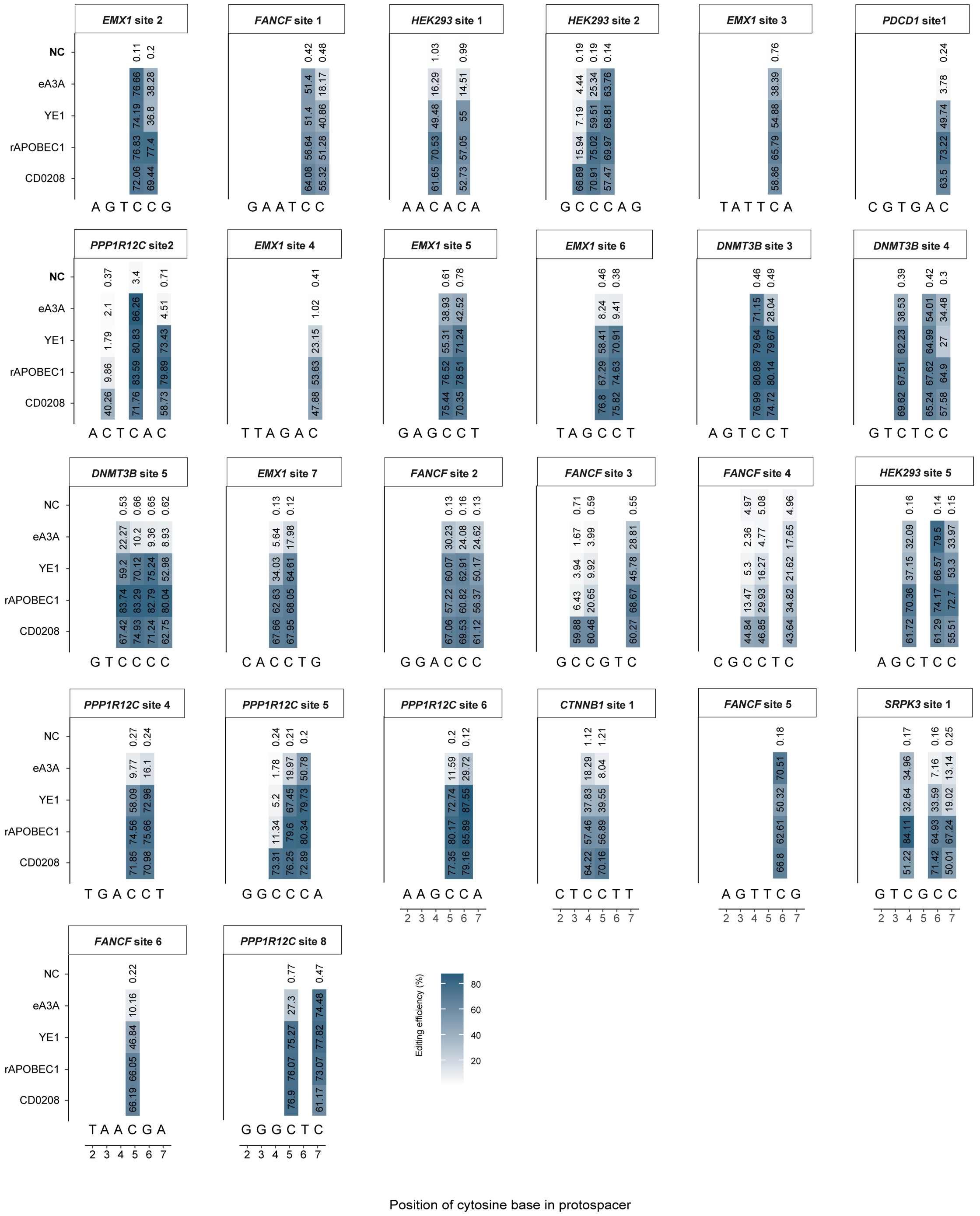

Supplementary Fig. 8 | Editing efficiency of CD0208-derived CBE. The on-target activity of the CD0208-derived CBE was tested at positions 3-7 nt for 26 endogenous target sites in HEK293T cells, with deaminases eA3A, YE1, and rAPOBEC1 serving as controls. The data represents the mean of three independent experiments.

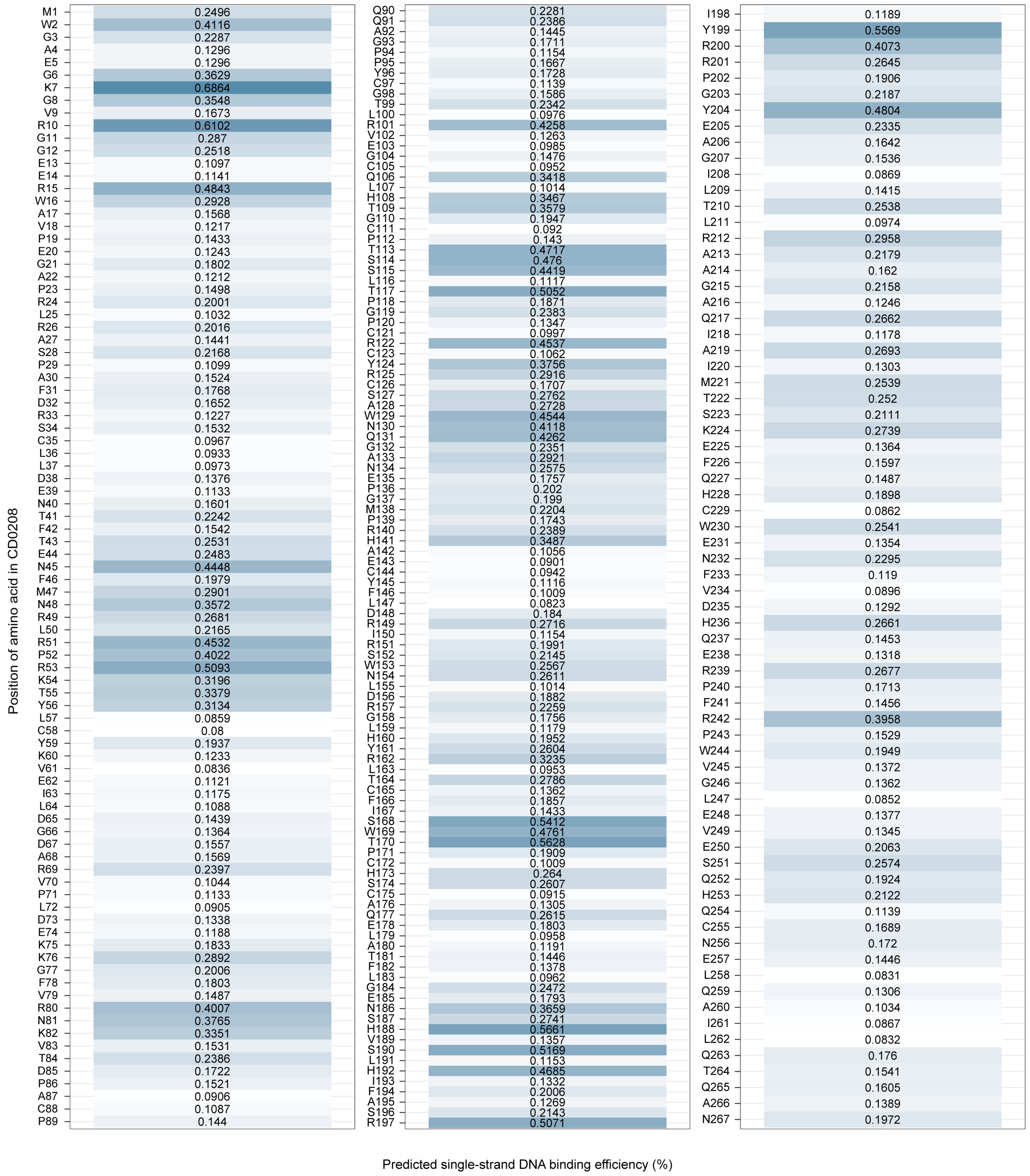

Supplementary Fig. 9 | Amino acid residues in CD0208 potentially interacted with ssDNA. Amino acid residues in the interaction between CD0208 and ssDNA were predicted using the DRNApred online software. The blue-to-white gradient indicates the predicted binding capacity.

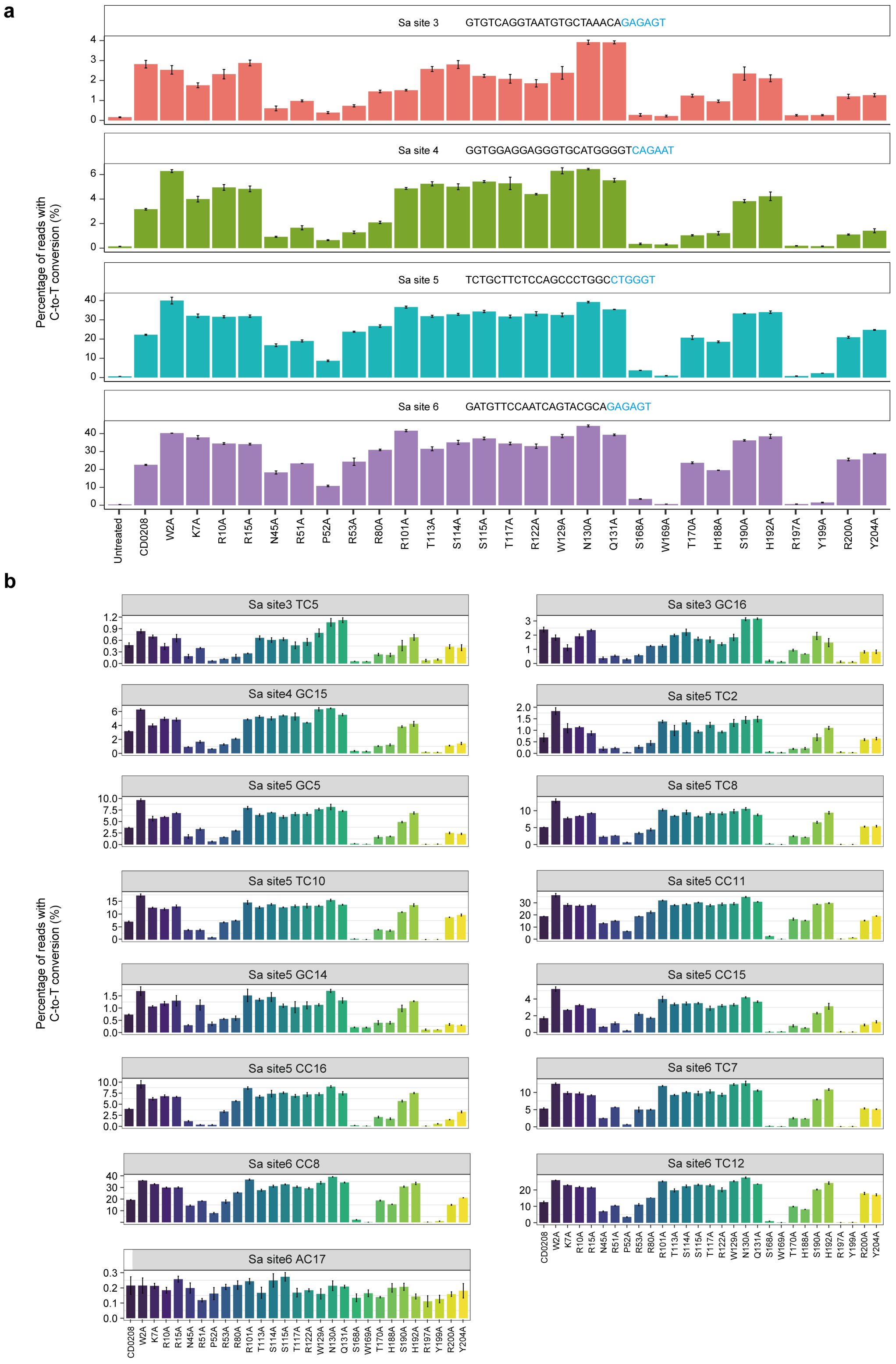

Supplementary Fig. 10 | Off-target effects of CD0208 variants-derived CBEs. a, The off-target effects of CD0208 variant CBEs using an orthogonal R-loop assay at four dSaCas9-sgRNA recognition sites (Sa site 3, Sa site 4, Sa site 5, and Sa site 6). **b**, C-to-T conversion of a single cytosine and its sequence context. Error bars indicate the mean ± SE of three independent experiments.

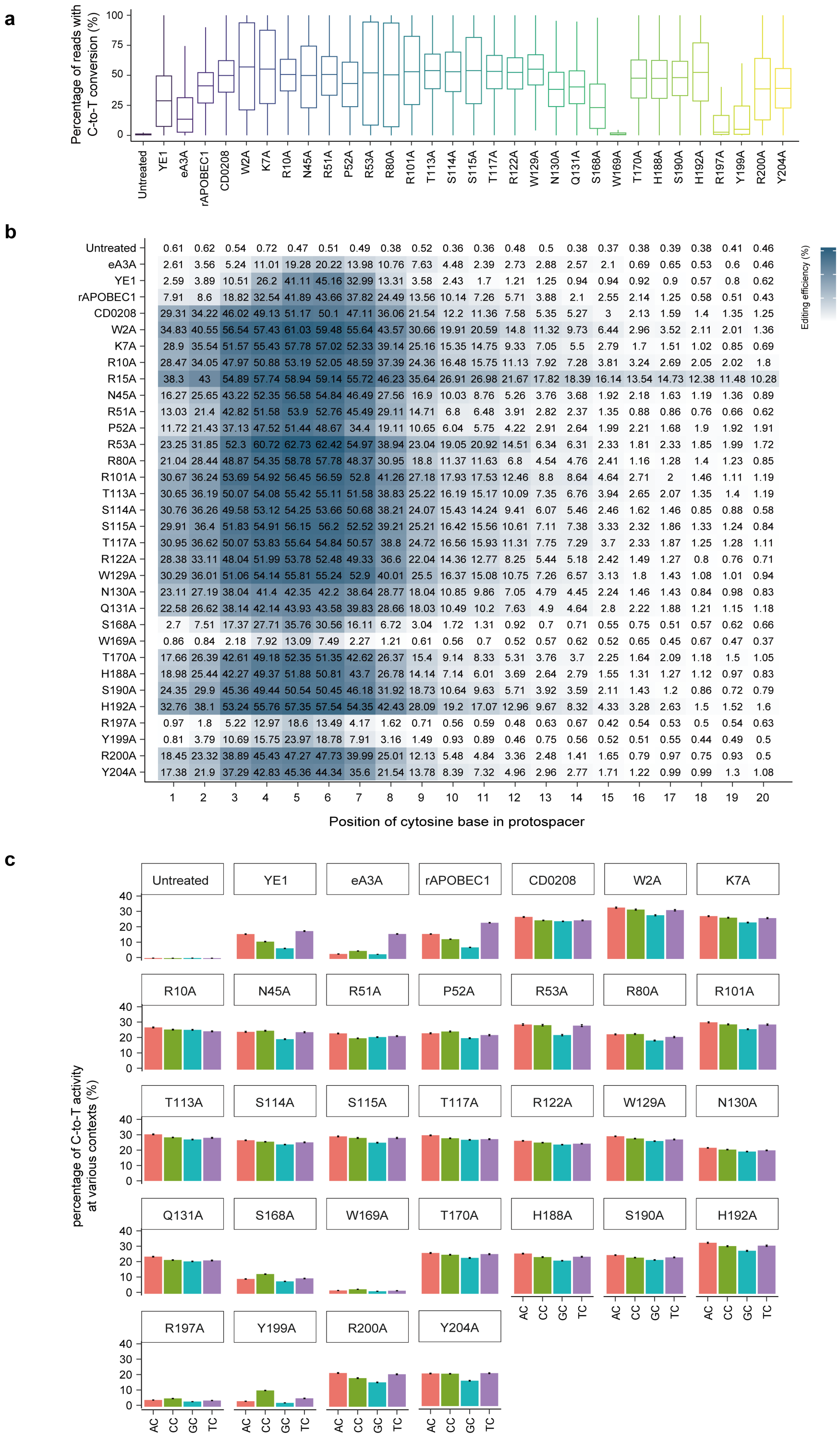

Supplementary Fig. 11 | Editing properties of CD0208 variants-derived CBEs. a, Editing efficiencies of CD0208 variants and three well-characterized deaminases (rAPOBEC1, YE1, and eA3A)-based CBEs in an 11,868 sgRNA-target library through deep sequencing analysis. **b**, Editing windows of CD0208 variants and three well-characterized deaminases (rAPOBEC1, YE1, and eA3A). The data represents the average editing efficiencies at 11,868 sgRNA-target sites. **c**, The sequence context preference of CBEs derived from CD0208 variants was compared with that of three well-characterized deaminases (rAPOBEC1, YE1, and eA3A). Error bars indicate the mean ± SE of the average editing efficiencies at 11,868 sgRNA-target sites.

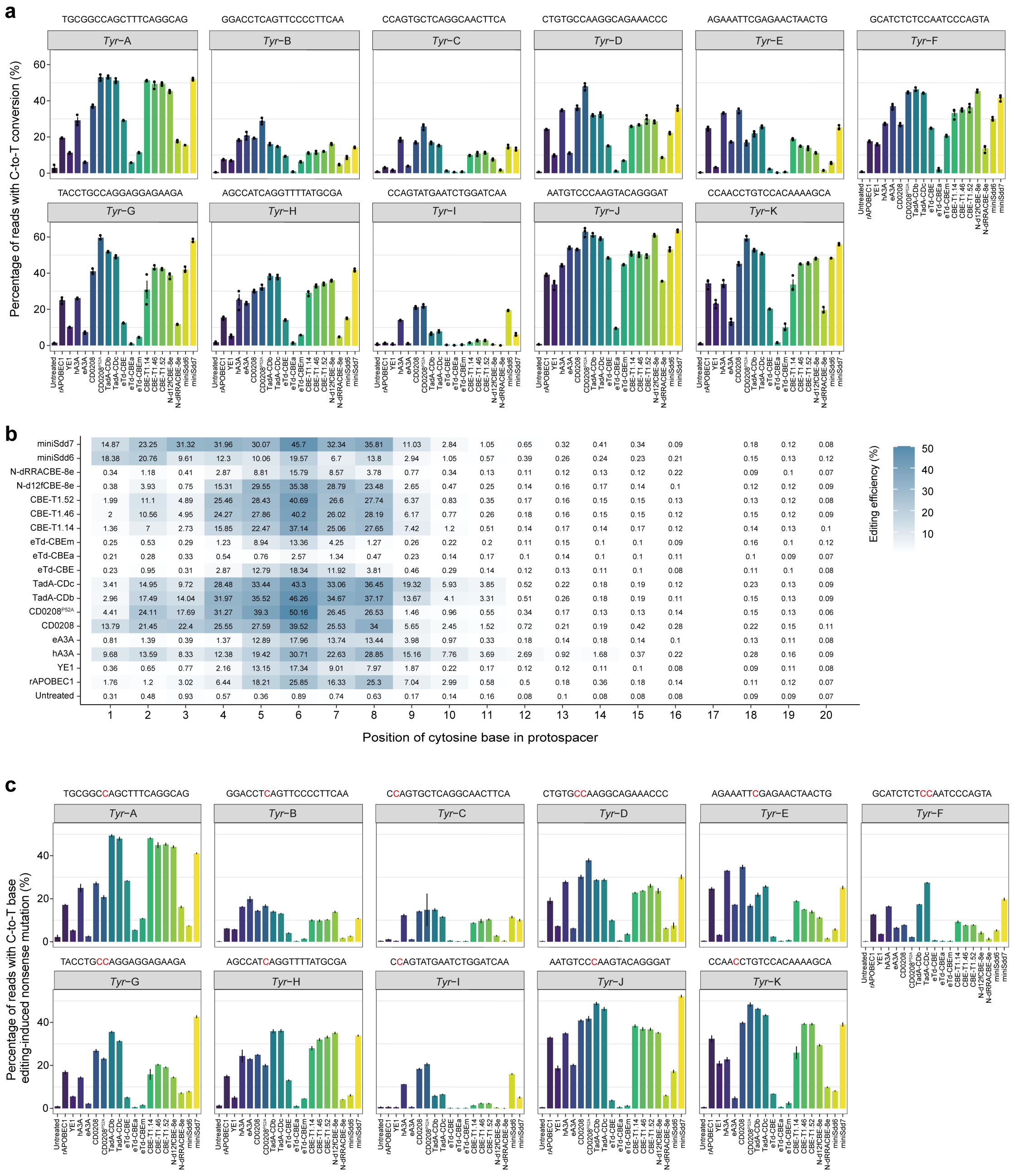

Supplementary Fig. 12 | CD0208^{P52A} CBE introducing nonsense mutations in the *Tyr* gene in mouse N2A cells. a, The C-to-T base editing efficiencies of CD0208^{P52A} CBE at 11 target sites in the *Tyr* gene in mouse N2A cells. The 17 classical and recently developed cytosine deaminase-derived CBEs serve as controls, including rAPOBEC1, YE1, hA3A, eA3A, CD0208, TadA-CDb, TadA-CDc, eTd-CBE, eTd-CBEa, eTd-CBEm, CBE-T1.14, CBE-T1.46, CBE-T1.52, N-d12fCBE-8e (28G46C), N-dRRACBE-8e (GGATY), miniSdd6, and miniSdd7. **b**, Editing windows of CD0208^{P52A} and 17 classical and recently developed cytosine deaminase-derived CBEs. **c**, Efficiency of the introduction of nonsense mutations at 11 target sites in the *Tyr* gene of N2A cells by CD0208^{P52A} CBE and 17 classical and recently developed cytosine deaminase-derived CBEs. Error bars indicate the mean ± SE of three independent experiments.

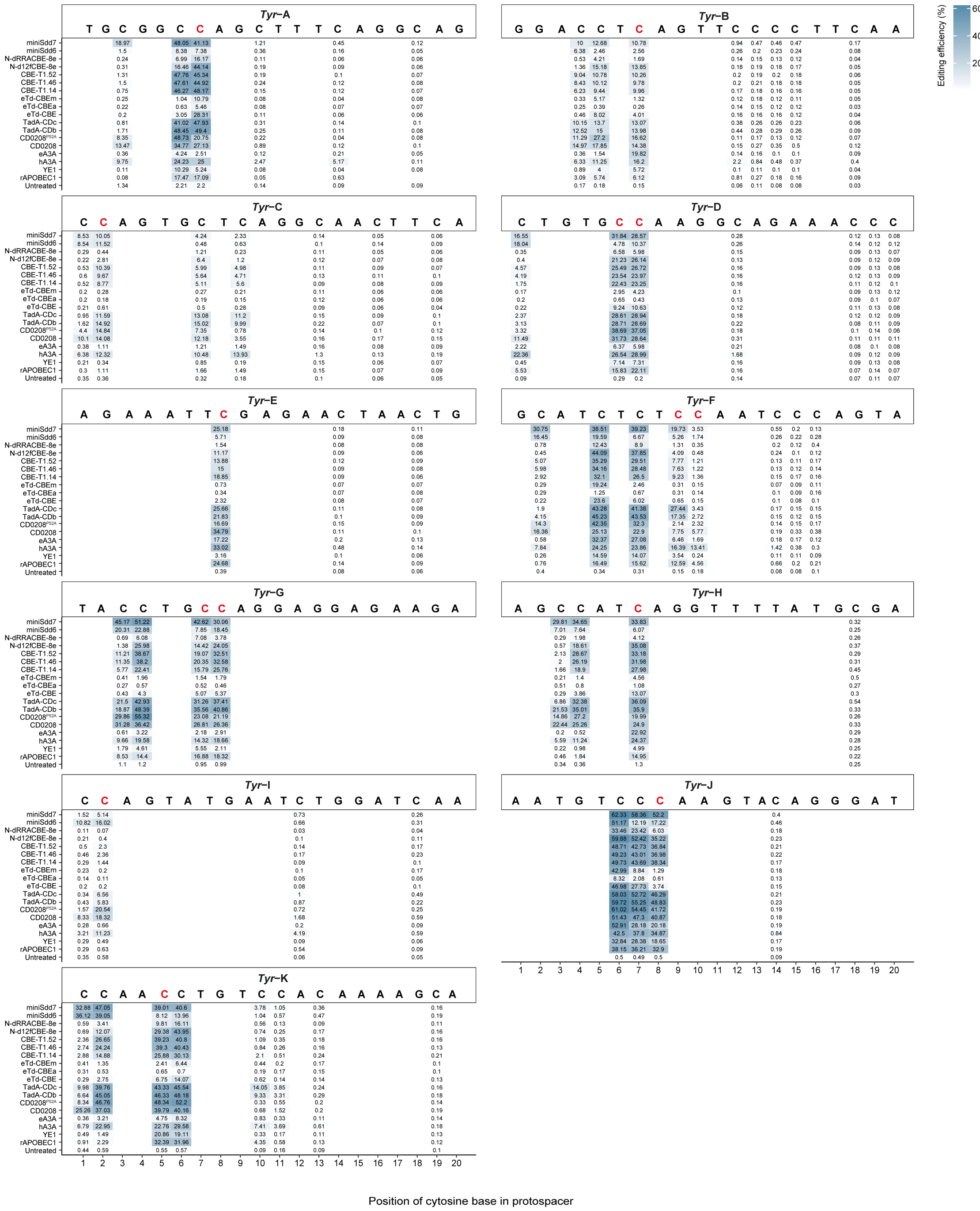

Supplementary Fig. 13 | Editing windows of CD0208^{P52A} CBE at 11 target sites in the *Tyr* gene in mouse N2A cells. The mentioned 17 classical and recently developed cytosine deaminase-derived CBEs serve as controls. The cytosine bases in red font indicate the site that is desired to be edited. The data represents the mean of three independent experiments.

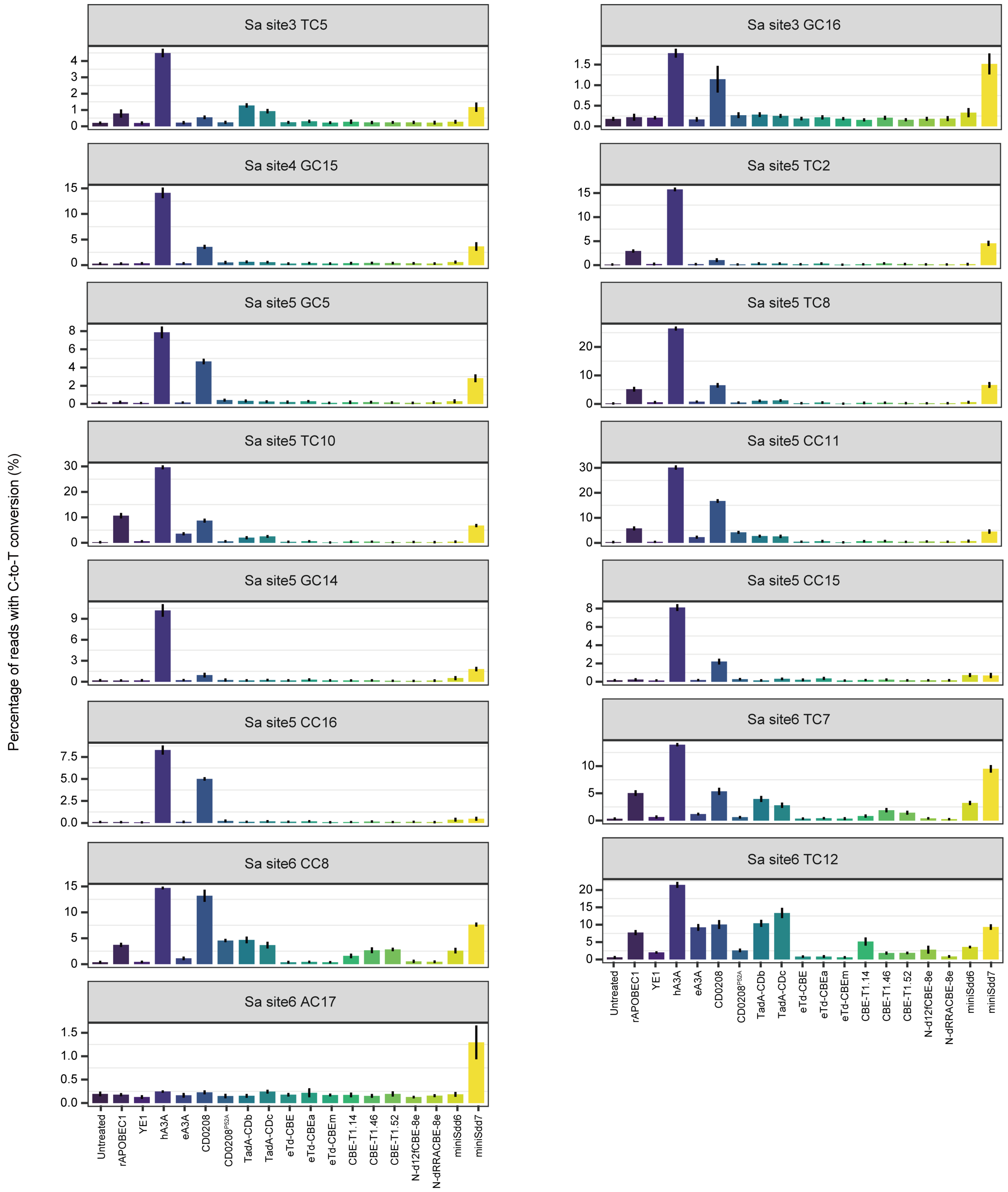

Supplementary Fig. 14 | Off-target effects of CD0208^{P52A}-derived CBE. Evaluation of the off-target effect of CD0208^{P52A} CBE using an orthogonal R-loop assay at four dSaCas9-sgRNA recognition sites (Sa site 3, Sa site 4, Sa site 5, and Sa site 6). The mentioned 17 classical and recently developed cytosine deaminase-derived CBEs serve as controls. Error bars indicate the mean ± SE of three independent experiments.

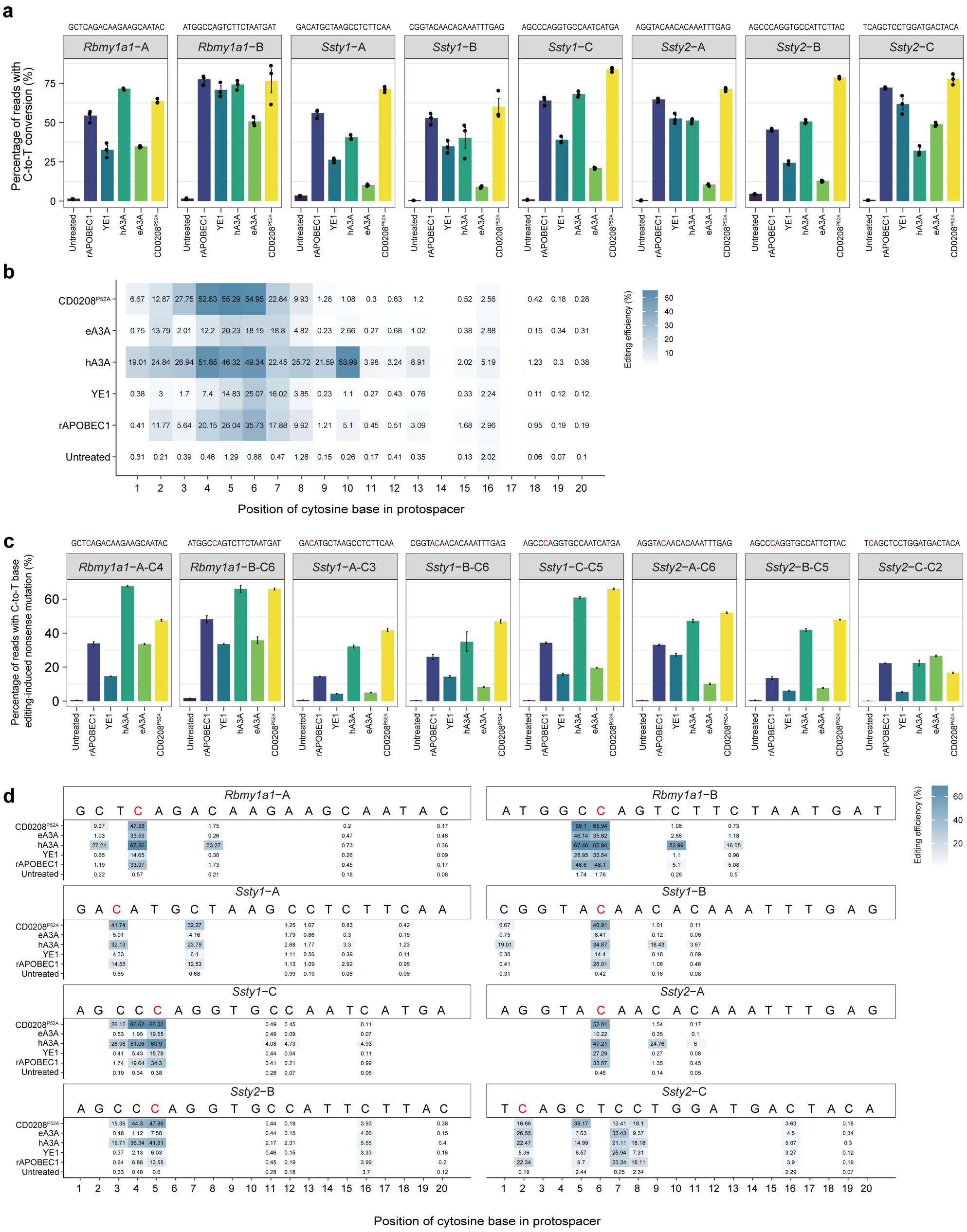

Supplementary Fig. 15 | CD0208^{P52A} CBE inducing nonsense mutations in three multi-copy genes in mESCs. a, Editing efficiency of CD0208^{P52A} CBE at eight target sites in three multi-copy genes (*Rbmy1a1, Ssty1, and Ssty2*) on the Y chromosome of mice in mESCs, with four well-characterized deaminase (hA3A, rAPOBEC1, YE1, and eA3A) -derived CBEs as controls. **b**, Editing windows of CD0208^{P52A}. **c**, Efficiency of introduction of nonsense mutation of CD0208^{P52A} at eight target sites. **d**, Editing windows of CD0208^{P52A}-derived CBEs at eight target sites. The cytosine bases in red font indicate the site that is desired to be edited. Error bars indicate the mean ± SE of three independent experiments.

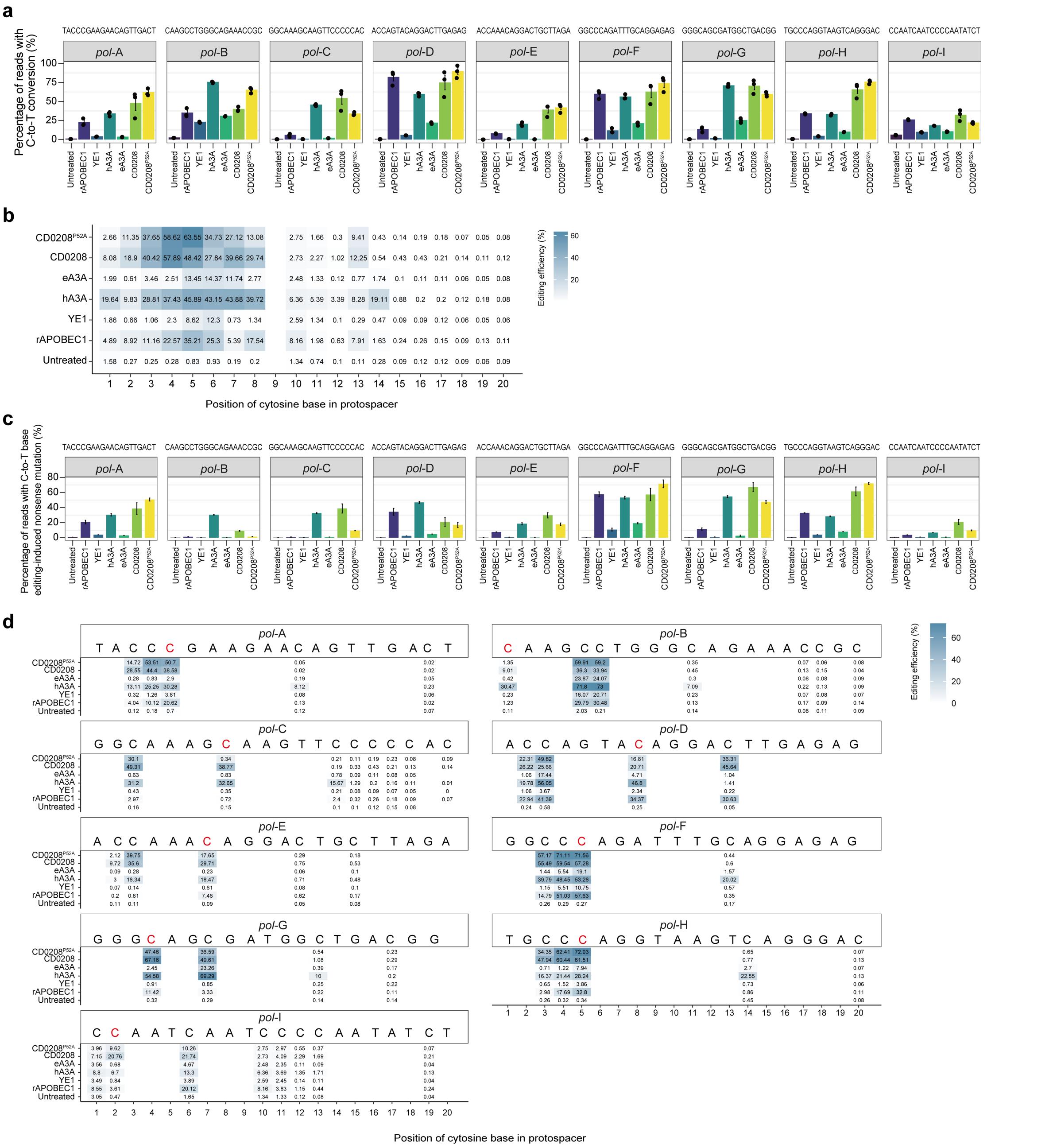

Supplementary Fig. 16 | CD0208^{P52A} CBE introducing nonsense mutations in muti-copy genes in porcine cell PK-15. a, Editing efficiency of CD0208^{P52A} CBE at nine target sites in the *pol* gene of PERV in PK-15 cells, CD0208 and four well-characterized deaminase (hA3A, rAPOBEC1, YE1, and eA3A) -derived CBEs as controls. **b**, Editing windows of CD0208^{P52A} and five deaminase-derived CBEs. **c**, Efficiency of introduction of nonsense mutation at nine target sites in the *pol* gene of PERV in PK-15 cells by CD0208^{P52A} CBE and five deaminase-derived CBEs. **d**, Editing windows of CD0208^{P52A} and five deaminase-derived CBEs at nine target sites. The cytosine bases in red font indicate the site that is desired to be edited. Error bars in **a**, and **c** indicate the mean ± SE of three independent experiments.

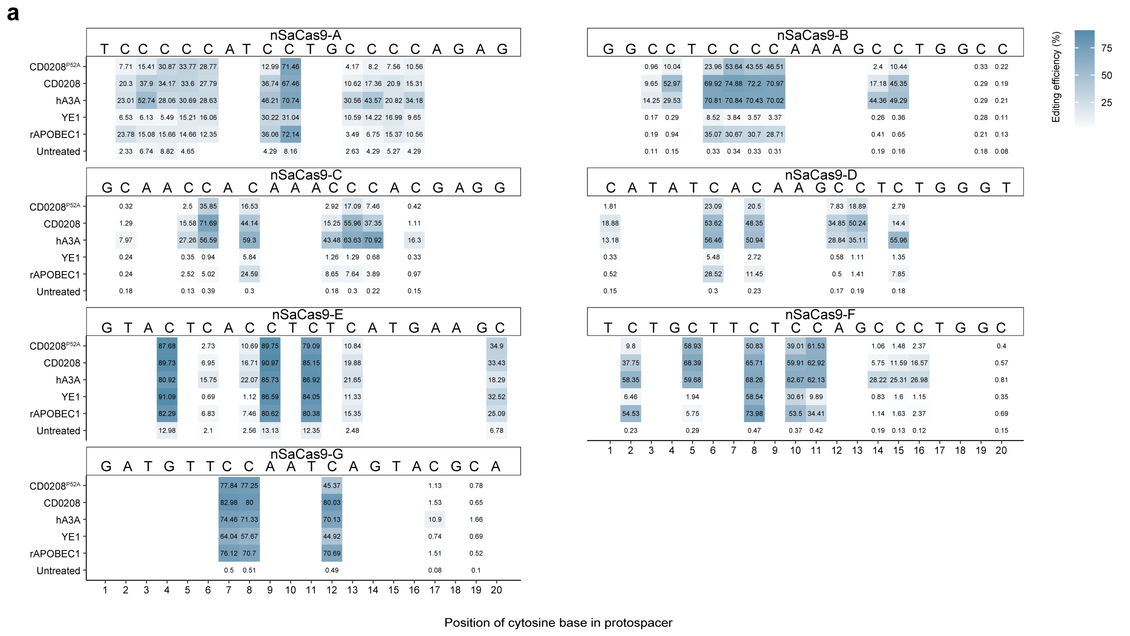

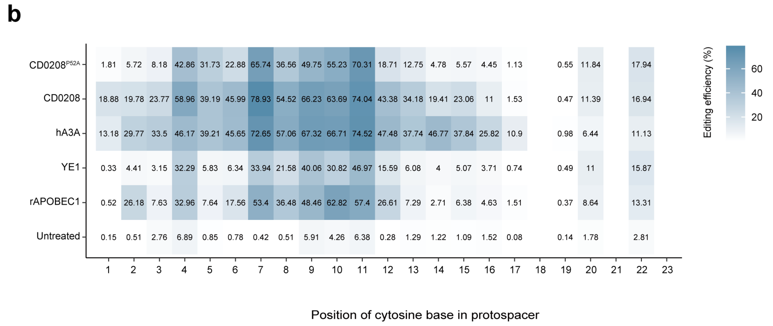

Supplementary Fig. 17 | Editing windows of CD0208 P52A-nSaCas9 CBE at seven target sites in HEK293T cells. a, Editing windows of CD0208P52A-nSaCas9 and four mentioned control CBEs at seven target sites in HEK293T cells. **b**, Summary of editing windows from **a**.

a

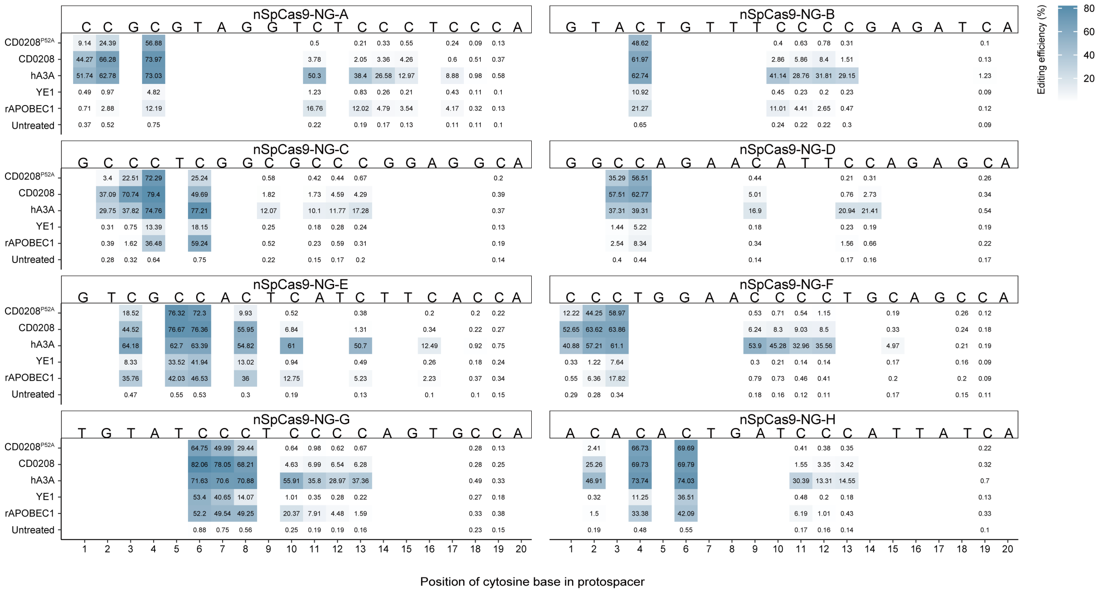

b

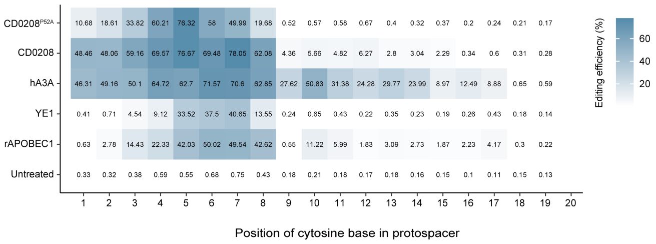

Supplementary Fig. 18 | Editing windows of CD0208^{P52A} nSpCas9-NG CBE at eight target sites in HEK293T cells. a, Editing windows of CD0208^{P52A} and and four deaminase (hA3A, rAPOBEC1, YE1, and CD0208) -derived nSpCas9-NG CBEs at eight target sites in HEK293T cells. **b**, Summary of editing windows from **a**.

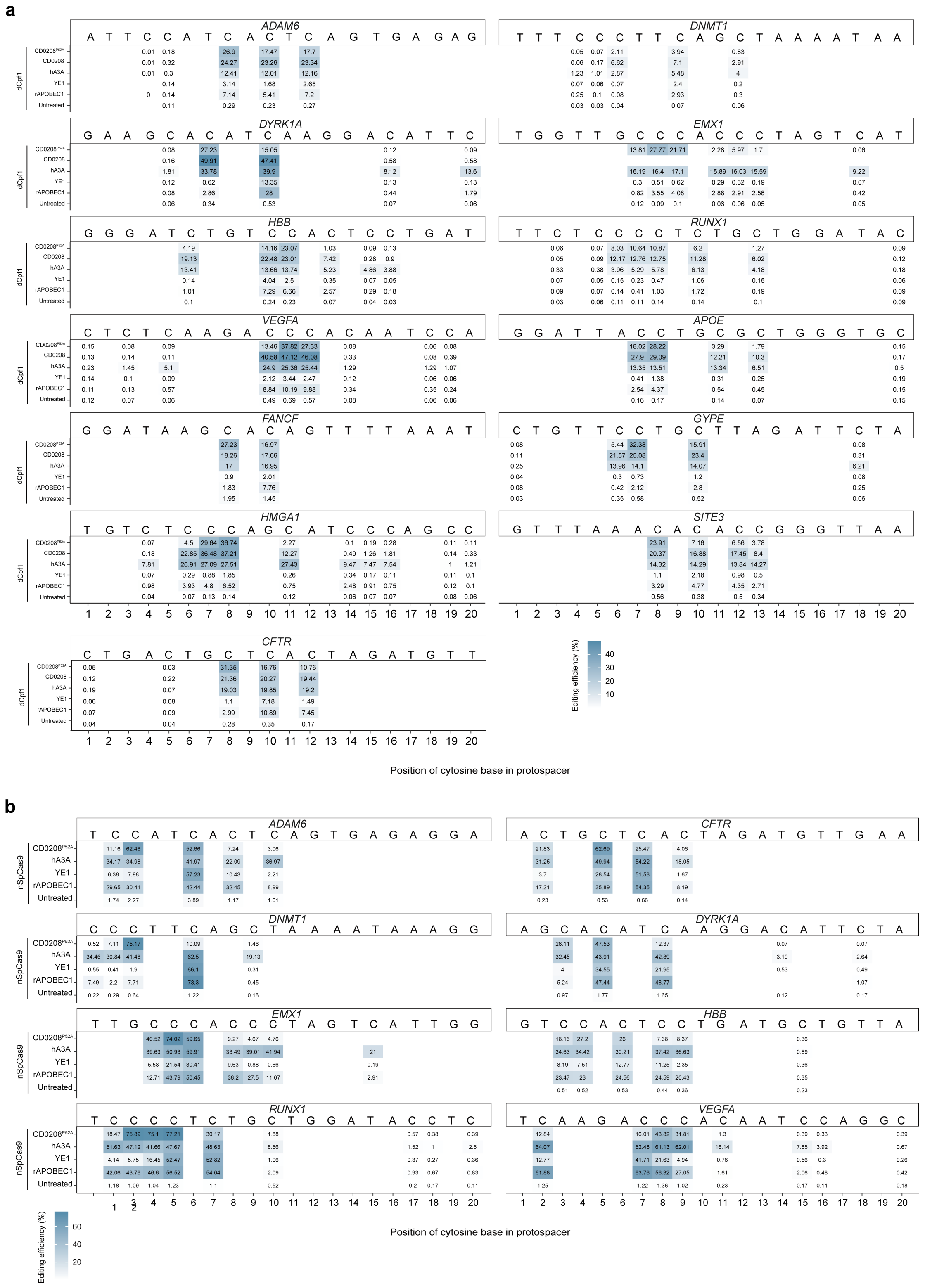

Supplementary Fig. 19 | Editing windows of CD0208^{P52A}-nSpCas9, and CD0208^{P52A}-dCpf1 CBEs at endogenous loci in HEK293T cells. a, Editing windows of CD0208^{P52A} and three deaminase (rAPOBEC1, YE1, and hA3A) -derived dCpf1 CBEs at 13 target sites in HEK293T cells. **b**, Editing windows of CD0208^{P52A} and three deaminase (rAPOBEC1, YE1, and hA3A) -derived nSpCas9 CBEs at eight target sites in HEK293T cells.

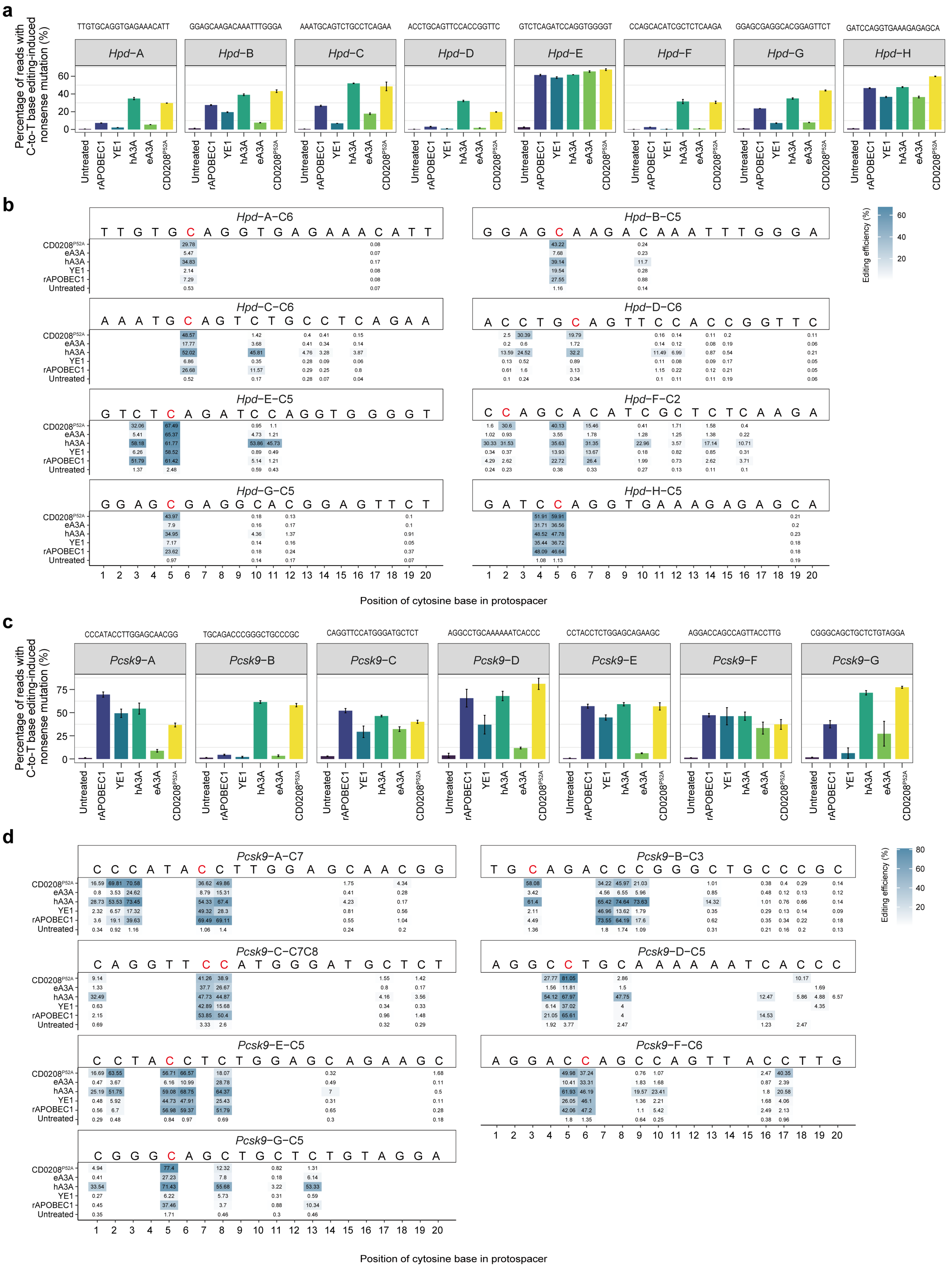

Supplementary Fig. 20 | CD0208^{P52A} CBE introducing nonsense mutations in *Hpd* and *Pcsk9* genes in N2A cells. a, Efficiency of introduction of nonsense mutation at eight target sites in *Hpd* gene in N2A cells by CD0208^{P52A} CBE and four deaminase (hA3A, rAPOBEC1, YE1, and eA3A) -derived CBEs. **b**, Editing windows of CD0208^{P52A} and four deaminase-derived CBEs at eight target sites in *Hpd* gene in N2A cells. The cytosine bases in red font indicate the site that is desired to be edited. **c**, Efficiency of introduction of nonsense mutation at seven target sites in *Pcsk9* gene in N2A cells by CD0208^{P52A} CBE and four deaminase-derived CBEs. **d**, Editing windows of CD0208^{P52A} and four deaminase-derived CBEs at seven target sites in *Pcsk9* gene in N2A cells. The cytosine bases in red font indicate the site that is desired to be edited. Error bars in **a**, and **c** indicate the mean ± SE of three independent experiments.
