# Supplementary material for: Structure-guided discovery of highly efficient cytidine deaminases with sequence-context independence
Source: Nat Biomed Eng. 2024 Jun 3;9(1):93–108. doi: 10.1038/s41551-024-01220-8 (PMC11754093; doi:10.1038/s41551-024-01220-8)
Supplement: Supplementary file 2 — Reporting Summary [file 41551_2024_1220_MOESM2_ESM.pdf]

nature portfolio

Corresponding author(s): Erwei Zuo

Last updated by author(s): Apr 18, 2024

Reporting Summary

Nature Portfolio wishes to improve the reproducibility of the work that we publish. This form provides structure for consistency and transparency in reporting. For further information on Nature Portfolio policies, see our Editorial Policies and the Editorial Policy Checklist.

Statistics

For all statistical analyses, confirm that the following items are present in the figure legend, table legend, main text, or Methods section.

n/a	Confirmed	
☐	☒	The exact sample size (n) for each experimental group/condition, given as a discrete number and unit of measurement
☐	☒	A statement on whether measurements were taken from distinct samples or whether the same sample was measured repeatedly
☐	☒	The statistical test(s) used AND whether they are one- or two-sided<br>*Only common tests should be described solely by name; describe more complex techniques in the Methods section.*
☒	☐	A description of all covariates tested
☒	☐	A description of any assumptions or corrections, such as tests of normality and adjustment for multiple comparisons
☐	☒	A full description of the statistical parameters including central tendency (e.g. means) or other basic estimates (e.g. regression coefficient) AND variation (e.g. standard deviation) or associated estimates of uncertainty (e.g. confidence intervals)
☐	☒	For null hypothesis testing, the test statistic (e.g. F, t, r) with confidence intervals, effect sizes, degrees of freedom and P value noted<br>*Give P values as exact values whenever suitable.*
☒	☐	For Bayesian analysis, information on the choice of priors and Markov chain Monte Carlo settings
☒	☐	For hierarchical and complex designs, identification of the appropriate level for tests and full reporting of outcomes
☒	☐	Estimates of effect sizes (e.g. Cohen's d, Pearson's r), indicating how they were calculated

Our web collection on statistics for biologists contains articles on many of the points above.

Software and code

Policy information about availability of computer code

Data collection	The qPCR reaction was performed on a CFXOplus96 System (BIO-RAD). High-throughput sequencing was collected and de-multiplexed via the Illumina NovaSeq or DNBSEQ-T7 sequencing platform. Flow-cytometry data were collected via a BD FACSAria III platform.
Data analysis	Sequencing reads were demultiplexed using fastq-multx (v1.4.2) and FASTQ files were analyzed using CRISPResso2 (v2.2.7). Phangorn package was utilized for the UPGMA phylogenetic tree. The 3D structural similarity matrix was constructed using pheatMap (v1.0.12). The phylogenetic tree was drawn using the ggtree (v3.8.0) script in R (v4.1). Article images were also drawn using R scripts such as ggplot2 (v3.4.2) and pheatMap. Flow cytometry data were analyzed using the FlowJo V10 software (TreeStar Inc.).

For manuscripts utilizing custom algorithms or software that are central to the research but not yet described in published literature, software must be made available to editors and reviewers. We strongly encourage code deposition in a community repository (e.g. GitHub). See the Nature Portfolio guidelines for submitting code & software for further information.

Materials & experimental systems

n/a	Involved in the study
☒	☐ Antibodies
☐	☒ Eukaryotic cell lines
☒	☐ Palaeontology and archaeology
☒	☐ Animals and other organisms
☒	☐ Clinical data
☒	☐ Dual use research of concern
☒	☐ Plants

Methods

n/a	Involved in the study
☒	☐ ChIP-seq
☐	☒ Flow cytometry
☒	☐ MRI-based neuroimaging

Eukaryotic cell lines

Policy information about cell lines and Sex and Gender in Research

Cell line source(s)	HEK293T cells and N2A cells were obtained from the cell bank of the Shanghai Institute of Biochemistry and Cell Biology, Chinese Academy of Sciences. PK-15 cells were purchased from ATCC (CCL-33). HepG2 cells were purchased from Procell Life Science & Technology (CL-0103).
Authentication	The cell lines were authenticated with STR profiling by the supplier.
Mycoplasma contamination	The cell lines were tested, and no contamination by mycoplasma was found.
Commonly misidentified lines (See ICLAC register)	No commonly misidentified cell lines were used.

Flow Cytometry

Plots

Confirm that:

☒ The axis labels state the marker and fluorochrome used (e.g. CD4-FITC).

☒ The axis scales are clearly visible. Include numbers along axes only for bottom left plot of group (a 'group' is an analysis of identical markers).

☒ All plots are contour plots with outliers or pseudocolor plots.

☒ A numerical value for number of cells or percentage (with statistics) is provided.

Methodology

Sample preparation	Cell-culture and transfection procedures are described in Methods. Briefly, the cells were washed and filtered through a 45-µm cell strainer cap before sorting.
Instrument	BD FACSAria III
Software	FlowJo X 10.0.7
Cell population abundance	Samples were found to be >95% pure when assessed with a second round of flow-cytometry and fluorescence-microscopy analysis.
Gating strategy	Positive boundaries were determined by GFP+ or mCherry+ cells, and negative boundaries were determined by non-transfected cells.

☐ Tick this box to confirm that a figure exemplifying the gating strategy is provided in the Supplementary Information.

Data

Policy information about availability of data

All manuscripts must include a data availability statement. This statement should provide the following information, where applicable:

- Accession codes, unique identifiers, or web links for publicly available datasets
- A description of any restrictions on data availability
- For clinical datasets or third party data, please ensure that the statement adheres to our policy

The raw sequence data are available from the National Center for Biotechnology Information (NCBI) Sequence Read Archive database under the accession code PRJNA1001278. All raw sequence data are also available from the China National GenBank DataBase (CNGBdb) under accession number CNP0004653. Source data are provided in this paper.

Research involving human participants, their data, or biological material

Policy information about studies with human participants or human data. See also policy information about sex, gender (identity/presentation), and sexual orientation and race, ethnicity and racism.

Reporting on sex and gender	The study did not involve human research participants.
Reporting on race, ethnicity, or other socially relevant groupings	–
Population characteristics	–
Recruitment	–
Ethics oversight	–

Note that full information on the approval of the study protocol must also be provided in the manuscript.

Field-specific reporting

Please select the one below that is the best fit for your research. If you are not sure, read the appropriate sections before making your selection.

☒ Life sciences ☐ Behavioural & social sciences ☐ Ecological, evolutionary & environmental sciences

For a reference copy of the document with all sections, see nature.com/documents/nr-reporting-summary-flat.pdf

Life sciences study design

All studies must disclose on these points even when the disclosure is negative.

Sample size	Sample sizes were determined on the basis of relevant published genome-editing experiments.
Data exclusions	No data were excluded.
Replication	All attempts at replication were successful.
Randomization	Owing to the small sample sizes, randomization was not relevant to the study. Covariates were controlled for by running controls in parallel whenever applicable.
Blinding	Blinding was not relevant to the study.

Reporting for specific materials, systems and methods

We require information from authors about some types of materials, experimental systems and methods used in many studies. Here, indicate whether each material, system or method listed is relevant to your study. If you are not sure if a list item applies to your research, read the appropriate section before selecting a response.
